# Supplementary material for: Fitness Trainers’ Educational Qualification and Experience and Its Association with Their Trainees’ Musculoskeletal Pain: A Cross-Sectional Study
Source: Sports (Basel). 2022 Aug 29;10(9):129. doi: 10.3390/sports10090129 (PMC9501401; doi:10.3390/sports10090129)
Supplement: Supplementary file 1 [file sports-10-00129-s001.zip › sports-1849803-supplementary.pdf]

**Supplementary Materials: Survey questionnaire**

**Title:** A study on fitness trainers' educational qualification and experience and its association with pain among their clients'

**Section A: Demographic data**

1. Name of the participant

2. Age___________________

3. Gender of the participant

1. Male 2. Female

4. Weight of the participant (in KG)

5. Height of the participant (in cm)

6. Occupation

1. Student 2. Employed 4. Homemaker 5. Unemployed

**Section B: Workout activity details of the participant**

7. Purpose of gym joining

1. Losing weight 2. Physical fitness 3. Bodybuilding 4. Recreation

8. How many hours do you spend working out in a day?

1. Half an hour 2. 1 hour 2. 2 hours 3. 3 hours or more

9. What kind of exercise do you perform at the gym (answer can be multiple)

- Aerobics
- Strength training
- Power training
- Muscle building

**Section C: Pain history**

10. Do you have any history of injury during a workout?

1. No 2. Once 3. Multiple time

If yes causes of that particular injury (answer can be multiple)

- Over-exercising
- Wrong holding
- Over weight lifting
- Lack of workout knowledge
- Fatigues
- Not cautious during exercise
- Inadequate information from Trainer

11. Do you currently have any pain? (Last 7 days)

1. Yes 2. No

If yes, locate the site of pain (can be multiple sites)

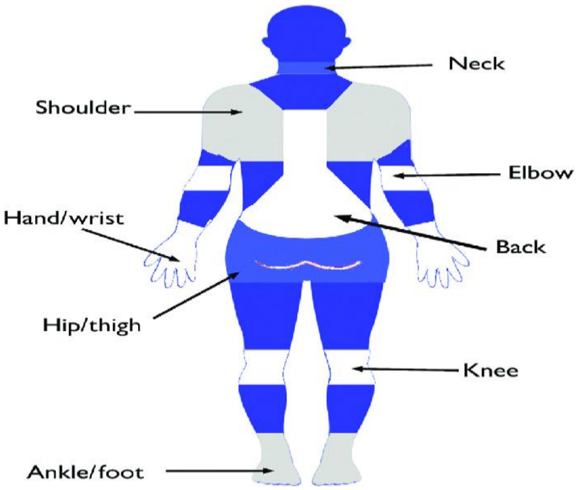

**Section D: Details about fitness trainer and center**

12. Name of the fitness center

13. Location of the center

1. Dhaka city corporation area 2. Outside Dhaka city corporation 3. Chittagong 4. Sylhet 5. Khulna

14. Degree of the fitness trainer

1. Certificate course 2. Diploma 3. Graduate 4. Post-graduate 5. No degree

15. Professional experience of the instructor

1. No experience 2. <1 year 3. 2-5 years 4. 6-10 years 5. >10 years
